# Clusters of lifestyle behaviours and their associations with socio-demographic characteristics in Dutch toddlers

**DOI:** 10.1007/s00394-022-03056-x

**Published:** 2022-11-24

**Authors:** Anne Krijger, Elly Steenbergen, Lieke Schiphof-Godart, Caroline van Rossum, Janneke Verkaik-Kloosterman, Liset Elstgeest, Sovianne ter Borg, Hein Raat, Koen Joosten

**Affiliations:** 1 Department of Pediatrics and Pediatric Surgery, Erasmus MC - Sophia Children’s Hospital, University Medical Center Rotterdam, P.O. box 2040, 3000 CA Rotterdam, The Netherlands; 2 Department of Public Health, Erasmus MC, University Medical Center Rotterdam, Rotterdam, The Netherlands; 3 National Institute for Public Health and the Environment, Bilthoven, The Netherlands; 4 Department of Medical Informatics, University Medical Center Rotterdam, Rotterdam, The Netherlands; 5 Reinier Academy, Reinier de Graaf Hospital, Delft, The Netherlands

**Keywords:** Lifestyle behaviour, Patterns, Overweight, Obesity, Diet, Physical activity

## Abstract

**Purpose:**

This study aimed to identify clusters of lifestyle behaviours in toddlers and assess associations with socio-demographic characteristics.

**Methods:**

We used data from the Dutch National Food Consumption Survey 2012–2016 and included 646 children aged 1–3 years. Based on 24-h dietary recalls and a questionnaire, a two-step cluster analysis was conducted to identify clusters in the intake of fruit, vegetables, sugar-sweetened beverages and unhealthy snacks, physical activity and screen time. Logistic regression models assessed associations between socio-demographic characteristics and cluster allocation.

**Results:**

Three clusters emerged from the data. The ‘relatively healthy cluster’ demonstrated a high intake of fruit and vegetables, low sugar-sweetened beverage and unhealthy snack intake and low screen time. The ‘active snacking cluster’ was characterised by high unhealthy snack intake and high physical activity, and the ‘sedentary sweet beverage cluster’ by high intake of sugar-sweetened beverages and high screen time. Children aged 1 year were most likely to be allocated to the ‘relatively healthy cluster’. Compared to children of parents with a high education level, children of parents with a low or middle education level were less likely to be in the ‘relatively healthy cluster’, but more likely to be in the ‘sedentary sweet beverage cluster’.

**Conclusion:**

Clusters of lifestyle behaviours can be distinguished already in children aged 1–3 years. To promote healthy lifestyle behaviour, efforts may focus on maintaining healthy behaviour in 1-year-olds and more on switching towards healthy behaviour in 2- and 3-year-olds.

**Supplementary Information:**

The online version contains supplementary material available at 10.1007/s00394-022-03056-x.

## Introduction

Overweight and obesity can occur as early as toddlerhood. Globally, 5.7% of children under 5 years were overweight or obese in 2020 [1]. This is a major public health concern as childhood obesity increases the risk of other (chronic) diseases, affecting both physical and mental health [2]. Moreover, childhood obesity often tracks into adulthood [3]. The main underlying cause of overweight and obesity lies in lifestyle behaviour, which may be established at a young age and likely persists as the child ages [2, 4, 5]. Unfavourable lifestyle behaviours, such as the intake of energy-dense, nutrient-poor foods, including sugar-sweetened beverages and snacks, as well as high levels of sedentary behaviour, are positively associated with obesity [6, 7]. Contrarily, diets characterised by high amounts of fruits and vegetables, and regular physical activity are associated with lower obesity risk [8, 9].

Many children do not meet the daily recommendations for dietary intake, physical activity and sedentary behaviour [10, 11]. However, children’s lifestyles can comprise both healthy and unhealthy behaviours simultaneously. Characterising lifestyle behaviour patterns in children can support the understanding of interrelationships (i.e. co-occurrence and interaction) between multiple lifestyle behaviours. Ultimately, this can contribute to developing guidelines and interventions that simultaneously address multiple unfavourable lifestyle behaviours in children.

Exploratory, data-driven techniques, such as cluster analysis and principal component analysis, can be used to gain insight into behaviour patterns [12]. Reviews of studies applying these methods to identify lifestyle behaviour clusters in children found that diet, physical activity and sedentary behaviour cluster in complex ways [13, 14]. In addition to clusters entirely characterised by healthy or unhealthy diets, physical activity and sedentary behaviours, clusters with a mixture of healthy and unhealthy behaviours have been commonly distinguished. To reach children most at risk of adverse health effects, it is essential to identify shared determinants of lifestyle behaviour clusters. As to determinants of lifestyle behavioural patterns in children, it has been shown that age, sex and socio-economic status (SES) are associated with lifestyle behaviour patterns [13, 14]. Lower SES, mostly indicated by parental education level, was found to be associated with unhealthier lifestyle patterns [13–15]. How other socio-demographic factors are associated with lifestyle behaviour patterns in children remains unclear.

To our knowledge, most studies on the clustering of lifestyle behaviours in children have been conducted in older children (≥ 5 years). Nevertheless, lifestyle habits develop early in life, and early identification of patterns and associated socio-demographic determinants might help to initiate timely interventions for modifying lifestyle behaviours when needed. Therefore, our study aims to identify clusters of co-occurring lifestyle behaviours, including intake of fruit, vegetables, sugar-sweetened beverages and unhealthy snacks, physical activity and screen time, and analyse their associations with socio-demographic characteristics in children aged 1–3 years who participated in the Dutch National Food Consumption Survey (DNFCS) 2012–2016.

## Methods

### Study population and data collection

We used data from the most recent DNFCS (2012–2016). The DNFCS is a recurrent survey on food and drinks consumption among the general Dutch population and specific subgroups. A detailed description of the DNFCS 2012–2016 has been published elsewhere [16]. Between November 2012 and January 2017, 6733 people aged 1–79 years were invited to participate in the study. Participants were drawn from market research consumer panels, representative for the Dutch population with regard to age, sex, education level (of the parents or caretakers for children up to 18 years), household region and household location urbanisation level. Data collection was completed for a set of 4313 participants, comprising 672 children aged 1–3 years. For the current study, we included children with complete data on all lifestyle behaviours of interest (*n* = 646). A flowchart of the study population selection is presented in Supplementary File 1.

An age-specific, general questionnaire completed by the parent(s) or caregiver(s) provided socio-demographic characteristics and information on lifestyle (e.g. amount of physical activity and electronic screen time) of the participating children. Dietary assessment was performed according to European Food Safety Authority (EFSA) guidelines [17]. Trained dieticians carried out two non-consecutive 24-h dietary recalls [19], equally spread across days of the week and seasons. The first 24-h dietary recall was conducted with a parent or caregiver during a home visit. The second 24-h dietary recall was completed by telephone about 4 weeks later. To adequately capture nutritional intake outside the home, for example at day care, both dietary recalls were combined with a food diary concerning the same day.

The Medical Ethical Committee of the University Medical Centre Utrecht approved the protocol and declared that the Dutch Medical Research Involving Human Subjects Act (WMO) was not applicable to the DNFCS 2012–2016 (reference number 12–359/C). Written informed consent was obtained from all parents/caregivers of participating children during the home visit.

### Lifestyle behaviours

#### Diet

The foods and drinks consumed as obtained by the 24-h dietary recalls were classified according to the food groups of the Dutch food-based dietary guidelines (‘Wheel of Five’ guidelines) [20]. Foods and drinks are categorised ‘within the Wheel of Five’ when consumption is advised by the Dutch food-based dietary guidelines and ‘outside the Wheel of Five’ when it is recommended to limit consumption of that particular food or drink. For the drinks category, for example, water and tea are categorised within the Wheel of Five, whereas sugar-sweetened beverages are not part of it. All sweet and savoury snacks, such as cookies, ice cream, and crisps, are categorised outside the Wheel of Five. We used the average intake of the two recall days per participant of the food groups fruit, vegetables, drinks outside the Wheel of Five (mainly sugar-sweetened beverages, therefore referred to as sugar-sweetened beverages in this paper) and snacks outside the Wheel of five (in this paper referred to as unhealthy snacks) in our analyses (g/day).

#### Physical activity

Time spent playing outside and participation in organised physical activity, such as swimming, toddler sports classes and dancing, was obtained from the general questionnaire. Parents or caregivers reported frequency of both activities on response categories ranging from ‘never/less than 1 day per week’ to ‘every day’. Response categories for average duration of playing outside ranged from ‘less than half an hour per day’ to ‘more than 3 h per day’. Average duration was converted from hours to minutes. Regarding organised physical activity, we translated one session as 60 min. We calculated the amount of physical activity (min/day) by the following equation: ((days playing outside * average duration of playing outside) + (days participating in organised physical activity * 60))/7.

#### Screen time

Time spent watching television or videos and using the computer or other types of electronic screens (such as a handheld game console or tablet) was also obtained from the general questionnaire. Frequency and average duration per session were reported by the parents on scales ranging from ‘never/less than 1 day per week’ to ‘every day’ and ‘less than half an hour per day’ to ‘more than 3 h per day’, respectively. Duration values were converted from hours to minutes. We calculated total screen time (min/day) by adding the amount of watching television/videos to the amount of computer/other screen use: ((days watching television * average duration of watching television) + (days using the computer * average duration of using the computer))/7.

### Socio-demographic characteristics

Information on age, sex, migration background, parental education level, and household size were obtained from the general questionnaire. Children’s migration background (Dutch, Western migration, non-Western migration) was defined based on the parents’ or caregivers’ country of birth. Children were assigned to the latter two categories when at least one parent or caregiver was born abroad [21]. Parental education level was divided into three categories (low, primary education, lower vocational education, advanced elementary education; middle, intermediate vocational education, higher secondary education; high, higher vocational education and university). The market research agency held data on household location region based on the Nielsen CBS division (west, north, east, south (of the Netherlands) and urbanisation level (strongly urbanised, > 1.500 addresses/km^2^; moderately urbanised, 1.000–1.500 addresses/km^2^; hardly urbanised, < 1.000 addresses/km^2^).

### Statistical analyses

All analyses were performed by using SPSS Statistics software (IBM SPSS Statistics for Windows, Version 25.0. Armonk, NY: IBM Corp.). Characteristics of the children were described in percentages and medians. After standardisation (by calculating Z-scores) of the lifestyle behaviour data, we performed a cluster analysis procedure comprising a hierarchical and consecutive non-hierarchical step. This cluster analysis approach was previously used by Fernández-Alvira et al. [22] and Yang et al. [23]. First, Ward’s method using squared Euclidean distance was applied to create initial cluster centres, with solutions ranging from two to six clusters. Thereafter, non-hierarchical k-means cluster analysis based on these cluster centres was conducted. The stability of the generated cluster solutions was examined by repeating the clustering procedure in a random sample of 50% of the study population and testing cluster allocation agreement by Cohen’s kappa. Mean values of lifestyle behaviours per cluster were described. Logistic regression models (univariable and multivariable) were used to calculate odds ratios (OR) for allocation to the generated clusters based on the socio-demographic determinants. We applied Bonferroni correction to adjust for multiple testing [*p* = 0.05/(number of clusters * number of socio-demographic characteristics)] [23].

#### Non-response analysis

Of the 672 children aged 1–3 years that participated in the DNFCS, children with missing data on the lifestyle behaviours of interest (*n* = 26) were compared (on lifestyle behaviours and socio-demographic characteristics) with children with complete data (*n* = 646) by using independent *t* tests and Chi-square tests.

## Results

### Population characteristics

The study sample included 646 children aged 1 (34.2%), 2 (31.0%) or 3 (34.8%) years, of which 49.7% were boys (Table 1). The majority of them were of Dutch origin (92.6%) and had parents with a high education level (66.7%). The most common household size consisted of four persons (43.5%). Participating children most often lived in the western part of the Netherlands (45.5%), which is analogous to a strongly urbanised household location (45.7%). The children consumed a median of 140 g (IQR 114) of fruit, 49 g (IQR 60) of vegetables, 362 g (340) of sugar-sweetened beverages, and 32 g (IQR 44) of unhealthy snacks per day. Further, they spent 54 (IQR 62) min/day on physical activity and used electronic screens for 39 min/day (IQR 78) (median values).

**Table 1:** Characteristics of children aged 1–3 years in the DNFCS 2012–2016 (*n* = 646)

Characteristic	Value
Age	
1 year	221 (34.2)
2 years	200 (31.0)
3 years	225 (34.8)
Sex (boys)	321 (49.7)
Migration background	
Dutch	598 (92.6)
Western migration	17 (2.6)
Non-Western migration	31 (4.8)
Parental education	
Low	27 (4.2)
Middle	188 (29.1)
High	431 (66.7)
Size of household	
Two or three persons	186 (28.8)
Four persons	281 (43.5)
Five or more persons	179 (27.7)
Region of household location	
West	294 (45.5)
North	75 (11.6)
East	146 (22.6)
South	131 (20.3)
Household location urbanisation level	
Strongly urbanised	295 (45.7)
Moderately urbanised	141 (21.8)
Hardly urbanised	210 (32.5)
Fruit intake (g/d)	140 (114)
Vegetable intake (g/d)	49 (60)
Sugar-sweetened beverage intake (g/d)	362 (340)
Unhealthy snack intake (g/d)	32 (44)
Duration of physical activity (min/d)	54 (62)
Duration of screen time (min/d)	39 (78)

Values are frequencies with percentages for categorical variables and medians with interquartile ranges for continuous variables

### Non-response analysis

Children with missing data on the lifestyle behaviours of interest (*n* = 26) all lacked data on physical activity only. These children did not differ with regard to the other lifestyle behaviours, nor in socio-demographic characteristics (for all, *p* > 0.05) with the children that had complete data (*n* = 646, data not shown).

### Cluster description

**Fig. 1:**
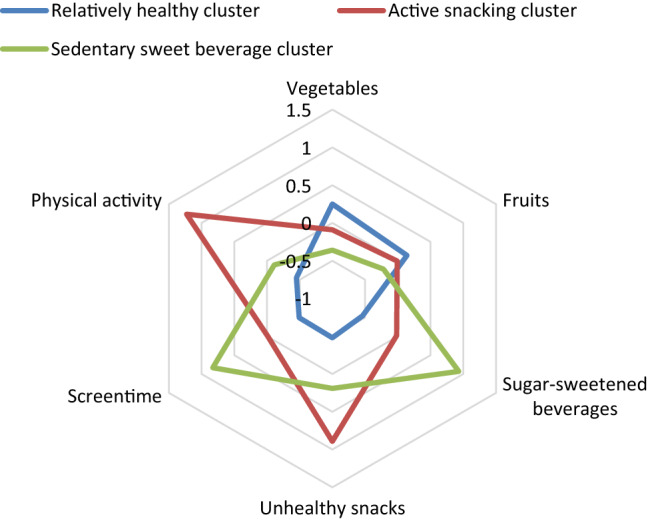
Z-scores of lifestyle behaviours in clusters of children aged 1–3 years in the DNFCS 2012–2016

Based on the dendrogram and highest Cohen’s kappa coefficient, a three-cluster solution based on the six lifestyle behaviours appeared to be the most accurate (*κ* = 0.937). Cluster 1 (comprising 49.7% of all children) was labelled the ‘relatively healthy cluster’ because compared to children in the other clusters, children in this cluster complied with guidelines relatively most [20, 24]. It was characterised by healthy dietary factors and low screen time as the Z-score was 0.14 (SE 0.05) for fruit intake, 0.25 (SE 0.06) for vegetable intake, – 0.54 (SE 0.03) for sugar-sweetened beverage intake, – 0.48 (SE 0.03) for unhealthy snack intake, and – 0.49 (SE 0.03) for screen time. High unhealthy snack intake (Z-score = 0.89, SE 0.11) and high physical activity (Z-score = 1.23, SE 0.09) were the main features of cluster 2, which was therefore labelled the ‘active snacking cluster’. Cluster 3 was mainly characterised by high intake of sugar-sweetened beverages (Z-score = 0.93, SE 0.07) and high screen time (Z-score = 0.83, SE 0.08) and was labelled ‘sedentary sweet beverage cluster’. The ‘relatively healthy cluster’ comprised 76% of the 1-year-olds. The mean age for the ‘relatively healthy cluster’ was 1.7 (SD 0.8) years and 2.3 (SD 0.7) years for the other two clusters (Table 2). Figure 1 demonstrates the lifestyle behaviour Z-scores of the various clusters in a radar chart.

**Table 2:** Lifestyle behaviours by clusters of children aged 1–3 years in the DNFCS 2012–2016

	Cluster 1 ‘relatively healthy cluster’^a^	Cluster 2 ‘active snacking cluster’^b^	Cluster 3 ‘sedentary sweet beverage cluster’^b^
	*N* = 321 (49.7%)	*N* = 135 (20.9%)	*N* = 190 (29.4%)
Age, y, mean (SD)	1.7 (0.8)	2.3 (0.7)	2.3 (0.7)
Fruit consumption, mean (SD)^c^	160 (81)	147 (103)	129 (83)
Z-score (SE)	0.14 (0.05)	–0.01 (0.10)	–0.22 (0.07)
Vegetable consumption, mean (SD)^c^	69 (51)	53 (44)	40 (34)
Z-score (SE)	0.25 (0.06)	–0.09 (0.08)	– 0.36 (0.05)
Sugar-sweetened beverage consumption, mean (SD)^c^	242 (174)	398 (225)	676 (298)
Z-score (SE)	–0.54 (0.03)	–0.02 (0.07)	0.93 (0.07)
Unhealthy snack consumption, mean (SD)^c^	24 (20)	72 (45)	47 (29)
Z-score (SE)	–0.48 (0.03)	0.89 (0.11)	0.19 (0.06)
Physical activity, mean (SD)^d^	44 (35)	133 (52)	63 (39)
Z-score (SE)	–0.45 (0.04)	1.23 (0.09)	–0.11 (0.05)
Screen time, mean (SD)^d^	24 (26)	48 (43)	90 (57)
Z-score (SE)	–0.49 (0.03)	–0.01 (0.07)	0.83 (0.08)

^a^Overall most consistent with national guidelines

^b^Named after most distinguishing lifestyle behaviours

^c^g/day

^d^Min/day

### Association between socio-demographic characteristics and cluster allocation

The ORs for cluster allocation based on the socio-demographic characteristics are presented in Table 3. Based on the three cluster solution, we used a Bonferroni adjusted *p* value of 0.003 [*p* = 0.05/(3*6)]. Children aged 1 year had higher odds for allocation to the ‘relatively healthy cluster’ than children aged 3 years old, with an OR of 7.48 (95% CI 4.91, 11.39; *p* < 0.001). Moreover, children aged 1 year had lower odds for allocation to the ‘active snacking cluster’ and ‘sedentary sweet beverage cluster’ compared to children aged 3 years, with ORs of 0.27 (95% CI 0.16, 0.46; *p* < 0.001) and 0.23 (95% CI 0.15, 0.37; *p* < 0.001), respectively. Compared to children of parents with a high education level, children of parents with a low education level had an OR of 0.06 (95% CI 0.01, 0.26; *p* < 0.001) for allocation to the ‘relatively healthy cluster’, and children of parents with a middle education level of 0.48 (95% CI 0.34, 0.68; *p* < 0.001). Contrarily, children of parents with a low education level had an OR of 6.71 (95% CI 2.92, 15.40; *p* < 0.001) for allocation to the ‘sedentary sweet beverage cluster’, and children of parents with a middle education level of 2.13 (95% CI 1.47, 3.08; *p* < 0.001), compared to children of parents with a high education level. We found no associations between parental education level and the ‘active snacking cluster’. Children from households of two or three persons had higher odds for the ‘relatively healthy cluster’ than children from four-person households, OR 1.87 (95% CI 1.28, 2.73, *p* = 0.001). This association disappeared in the multivariable model. Sex, migration background, region of household location, and household location urbanisation level were not associated with allocation to any cluster.

**Table 3:** Association of socio-demographic characteristics with clusters of children aged 1–3 years in the DNFCS

	Univariable models^a^			Multivariable models		
	‘Relatively healthy cluster’^c^ *N* = 321 OR (95% CI)	‘Active snacking cluster’^d^ *N* = 135 OR (95% CI)	‘Sedentary sweet beverage cluster’^d^ *N* = 190 OR (95% CI)	‘Relatively healthy cluster’^c^ *N* = 321 OR (95% CI)	‘Active snacking cluster’^d^ *N* = 135 OR (95% CI)	‘Sedentary sweet beverage cluster’^d^ *N* = 190 OR (95% CI)
Age						
1 year	7.48 (4.91,11.39)**	0.27 (0.16, 0.46)**	0.23 (0.15, 0.37)**	7.78 (4.92, 12.31)**	0.30 (0.17, 0.52)**	0.22 (0.14, 0.37)**
2 years	1.78 (1.19, 2.65)*	0.78 (0.50, 1.20)	0.70 (0.47, 1.04)	1.81 (1.18, 2.76)*	0.82 (0.53, 1.28)	0.67 (0.44, 1.02)
3 years	Ref	Ref	Ref	Ref	Ref	Ref
**Sex**						
Girl	Ref	Ref	Ref	Ref	Ref	Ref
Boy	1.01 (0.74, 1.38)	1.16 (0.79, 1.69)	0.88 (0.63, 1.23)	1.00 (0.70, 1.43)	1.23 (0.83, 1.84)	0.82 (0.57, 1.18)
Migration background						
Dutch	Ref	Ref	Ref	Ref	Ref	Ref
Western migration	2.57 (0.89, 7.37)	0.22 (0.03, 1.66)	0.74 (0.24, 2.29)	3.42 (1.13, 10.39)*	0.19 (0.02, 1.44)	0.67 (0.21, 2.18)
Non-Western migration	1.94 (0.92, 4.13)	0.12 (0.02, 0.86)*	1.14 (0.53, 2.47)	1.50 (0.63, 3.60)	0.16 (0.02, 1.18)	1.62 (0.69, 3.79)
Parental education						
Low	0.06 (0.01, 0.26)**	1.40 (0.58, 3.43)	6.71 (2.92, 15.40)**	0.06 (0.01, 0.27)**	1.03 (0.40, 2.64)	5.91 (2.47, 14.11)**
Middle	0.48 (0.34, 0.68)**	1.15 (0.76, 1.75)	2.13 (1.47, 3.08)**	0.41 (0.28, 0.61)**	1.18 (0.76, 1.82)	2.21 (1.50, 3.26)**
High	Ref	Ref	Ref	Ref	Ref	Ref
Size of household						
Two or three persons	1.87 (1.28, 2.73)**	0.55 (0.33, 0.91)*	0.71 (0.47, 1.08)	1.18 (0.76, 1.83)	0.73 (0.42, 1.24)	1.03 (0.65, 1.65)
Four persons	Ref	Ref	Ref	Ref	Ref	Ref
Five or more persons	0.78 (0.53, 1.14)	1.29 (0.84, 2.00)	1.07 (0.72, 1.60)	0.65 (0.42, 1.00)	1.32 (0.84, 2.08)	1.21 (0.78, 1.87)
Region of household location						
West	Ref	Ref	Ref	Ref	Ref	Ref
North	0.64 (0.38, 1.07)	1.13 (0.60, 2.10)	1.51 (0.89, 2.58)	0.70 (0.37, 1.29)	0.88 (0.44, 1.75)	1.55 (0.84, 2.86)
East	0.91 (0.61, 1.35)	1.26 (0.78, 2.04)	0.93 (0.59, 1.45)	1.05 (0.66, 1.68)	1.11 (0.66, 1.88)	0.87 (0.53, 1.44)
South	0.84 (0.56, 1.27)	1.13 (0.68, 1.88)	1.12 (0.71, 1.75)	1.04 (0.64, 1.70)	0.96 (0.56, 1.64)	1.00 (0.61, 1.64)
Household location urbanisation level						
Strongly urbanised	Ref	Ref	Ref	Ref	Ref	Ref
Moderately urbanised	0.87 (0.58, 1.30)	1.03 (0.62, 1.72)	1.16 (0.75, 1.79)	1.12 (0.70, 1.78)	0.88 (0.51, 1.50)	1.05 (0.65, 1.69)
Hardly urbanised	0.71 (0.50, 1.02)	1.47 (0.96, 2.26)	1.10 (0.74, 1.62)	0.90 (0.58, 1.40)	1.23 (0.76, 2.00)	0.96 (0.60, 1.51)

Values are ORs with 95% CI, calculated by using logistic regression

The * and ** indicate the significance level

Statistically significant values are highlighted in bold

^a^In the univariable models, each independent variable was entered separately

^b^In the multivariable models, all independent variables were entered simultaneously

^c^Overall most consistent with national guidelines

^d^Named after most distinguishing lifestyle behaviours *p < 0.05, **p < 0.003 (Bonferroni-corrected *p* value)

## Discussion

We aimed to identify clusters of lifestyle behaviours in Dutch children aged 1–3 years and assess associations with socio-demographic characteristics. Three distinct lifestyle clusters emerged from the data: the ‘relatively healthy cluster’, ‘active snacking cluster’ and ‘sedentary sweet beverage cluster’. The socio-demographic factors age, parental education level and household size were associated with cluster allocation. We found no associations with sex, migration background, region of household location and household location urbanisation level.

In accordance with our findings, previous studies demonstrated healthy, unhealthy and mixed clusters in children [13, 14]. However, precise results differ, partly due to differences in the behaviours considered and in behavioural assessment and clustering techniques. Gubbels et al. and Wang et al. also examined clustering of lifestyle behaviours in Dutch toddlers and identified two and three clusters, respectively [25, 26]. Among 2-year-olds, a ‘sedentary snacking cluster’, characterised by high screen time and high intake of unhealthy snacks and drinks, and a ‘fibre cluster’, mainly depicted by high intakes of fruit, vegetables and brown bread, and low white bread intakes, emerged [25]. Clusters labelled as ‘unhealthy lifestyle pattern’, ‘low snacking and low screen time pattern’, and ‘active, high fruit and vegetable, high snacking and high screen time pattern’ were distinguished among 3-year-olds [26]. Similar to these Dutch studies [25, 26] and to results from other countries [4, 27, 28], we demonstrated that high screen time levels often cluster with high consumption of energy-dense products. Studies in children 5 years and older have suggested that screen time activities, such as watching TV, act as a conditioned cue to drink or eat and distract from feelings of satiety, which might be the two most important underlying mechanisms [29]. In addition, unhealthy food advertisements on TV, computer or other electronic screens may enhance this consumption behaviour [30]. Our other cluster demonstrated high physical activity co-occurring with high intake of unhealthy snacks. This was previously also found in Dutch children of 6 years old [23]. One could argue that parents offer their child a snack as a reward or energy replenishment after physical activity; however, possible explanations need to be further elucidated.

Children aged 1 year were most likely to be allocated to the ‘relatively healthy cluster’. As 1-year-olds have not been included in previous cluster analyses, this is a novel finding. Nevertheless, there are several reasons why lifestyle behaviour in this age group might differ from those of 2- and 3-year-olds. Children aged 1 year have just transitioned from breast or bottle feeding and complementary foods to the family meal time routine. One could argue that parents are, therefore, still conscious of their child’s diet, which is reflected in a relatively higher intake of fruit and vegetables and lower intake of sugar-sweetened beverages and unhealthy snacks. This reason, more focus and consciousness, may also be underlying the fact that children from a household with two or three persons—and therefore most likely one child—had higher odds for allocation to the ‘relatively healthy cluster’. The absence of an association with household size in the multivariable model argues that another factor, possibly age, plays an underlying role. Children aged 1 year might also be more accepting of the (healthy) food their parents offer and most likely will not ask for unhealthy snacks, sugar-sweetened beverages or screen time themselves. They might also consume less of those unhealthier foods because of their lower nutritional needs and longer sleep duration than children aged 2 and 3 years. We presume that the low amount of physical activity in the ‘relatively healthy cluster’ is an underestimation attributable to the physical activity items in the questionnaire. As forms of movement for children aged 1 year (e.g. creeping, crawling, floor play) had not been assessed in this questionnaire, the total amount of physical activity would probably have been greater. Nonetheless, as our results indicate that lifestyle behaviours are healthier in 1-year-olds than in 2- and 3-year-olds, preventive efforts should focus on preserving healthy behaviours in 1-year-old children, i.e. before unhealthy behaviours have rooted.

Although we have to be careful with strong statements given the small group of parents with a low education level, our results support previous studies that have shown that a lower parental education level is associated with clusters comprising less healthy behaviours in young children [4, 23, 25–28]. It seems possible that lower-educated parents possess less knowledge about healthy lifestyle habits for their children or that parenting practices and food environment mediate this association [31–33]. Howbeit, as parents play a crucial role in providing and controlling food and activity habits of children aged 1–3 years, interventions aimed at improving these habits should be tailored to the needs of parents with lower education levels.

### Strengths and limitations

Dietary assessment through 24-h dietary recalls is a major strength of our study, as it does not alter food consumption and has an infinite degree of specificity of the foods consumed. In addition, 24-h dietary recalls are sensitive to culture-specific differences and, when repeatedly conducted, can capture habitual dietary habits. The young age of the study participants, especially 1-year-olds, is another asset and adds new evidence to the importance of early preventive health care.

The young age of the participants might also be a limitation, as age might have been the most important factor in distinguishing lifestyle clusters. Furthermore, it was technically impossible to calculate the exact habitual intake for every individual separately. Therefore, we used the average intake of the two recall days per participant as a reflection of habitual intake, but we are aware that this method might be less accurate. Data on physical activity and screen time were obtained by means of categorical questions. Although included as continuous variables in our analyses, the results of physical activity and screen time, therefore, have limited precision, i.e. are accurate to half an hour. We also acknowledge the sample size as a limitation that may have hampered the robustness of the clusters identified and may have led to selection bias. The low number of participants of non-Dutch origin and from parents with a low education level is another limitation that possibly affected the reliability and generalisability of our results. Due to the cross-sectional design of the DNFCS, we could not draw causal conclusions on the association between cluster allocation and weight status. Besides, data was obtained between 2012 and 2017 and new ‘Wheel of Five’ guidelines have been published in the meantime, which may affect current dietary intake.

### Conclusions

We distinguished three clusters of lifestyle behaviours in children as young as 1–3 years of age. Children aged 1 year were more likely to be in the cluster that portrayed healthy behaviour than children aged 2 and 3 years, which suggests that maintaining healthy behaviour and changing towards more healthy behaviour should be promoted in these age groups, respectively. These preventive efforts should take parental education level into consideration. Future longitudinal research should assess cluster allocation evolution and its association with weight status.

## Supplementary Information

Below is the link to the electronic supplementary material.

Supplementary file1 (DOCX 48 KB)

## Data Availability

The data and code used in this study are available upon request from the corresponding author.
