# Supplementary figures and images for: Clusters of lifestyle behaviours and their associations with socio-demographic characteristics in Dutch toddlers
Source: Eur J Nutr. 2022 Nov 24;62(3):1143–51. doi: 10.1007/s00394-022-03056-x (PMC10030397; doi:10.1007/s00394-022-03056-x)

**Supplementary File 1**


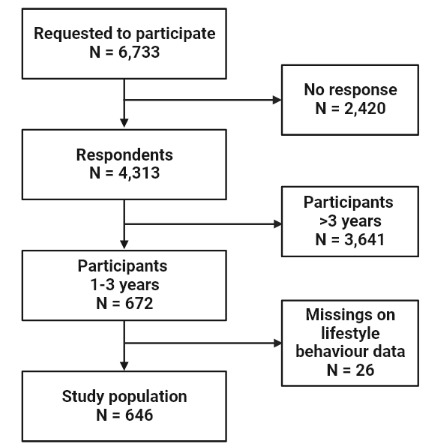


Supplementary Figure 1: Flowchart of study population selection

Supplement: Supplementary file 1 — Supplementary file1 (DOCX 48 KB) [file 394_2022_3056_MOESM1_ESM.docx]
